# Histopathological spectrum of duodenal polyps in a retrospective ten-year study

**DOI:** 10.15190/d.2022.2

**Published:** 2022-03-21

**Authors:** Varnika Rai, Anurag Saha, Ritu Verma, Vipul Jain, Bhanita Baro

**Affiliations:** ^1^Department of Pathology, Sanjay Gandhi Postgraduate Institute of Medical Science, Lucknow, Uttar Pradesh, India; ^2^Department of Pathology, Topiwala National Medical College and Nair Ch. Hospital, Mumbai, Maharashtra, India

**Keywords:** Upper gastrointestinal (UGI); Institution's Hospital Intranet System (HIS); Hematoxylin and eosin (H and E); Immunohistochemistry (IHC); End staged renal disease (ESRD); Peutz-jeghers (P-J); PTEN hamartoma tumour syndrome (PHTS); Familial adenomatous polyposis (FAP); Neuroendocrine tumour cases (NET); Gastrointestinal stromal tumor (GIST); Gangliocytic paragangliomas (GP); Duodenal lipomas (DL).

## Abstract

INTRODUCTION AND AIMS: Duodenal polyps are rare in patients undergoing upper gastrointestinal endoscopy. The present study is an experience of the histopathological spectrum of the duodenal polyps and its correlation with the clinical and endoscopic findings in a tertiary care centre. MATERIALS AND METHODS: The present study is a 10-year retrospective study from the year 2011 to 2020. All the relevant clinical, endoscopic and radiologic findings were retrieved from the hospital medical records. Old histopathology slides were restained, and wherever required, special stains and immunohistochemistry (IHC) were performed. All the cases were reviewed. The present study mainly included descriptive statistics with categorical and continuous variables.RESULTS: Total 81 cases of duodenal polyps were studied in the period of 10 years. The median age was 48 years. Male: female ratio was 2.2:1. The most common presenting system was abdominal pain. We experienced both solitary and multiple polyps. The majority of the duodenal polyps were non-neoplastic, with unremarkable mucosa or inflammatory type. Unlike previous studies the most common site for the hyperplastic polyp in the present study was the first part of the duodenum. Among the neoplastic polyps, adenomatous polyp was the most common type. Contrary to the previous studies, our study showed the first part of the duodenum as the most common site for the sporadic nonampullary adenomatous duodenal polyps. Of the rare entities, we encountered a single case each of lipomatous polyp and gangliocytic paraganglioma. Among the syndromes we encountered two cases of Peutz-Jeghers syndrome and one case of familial adenomatous polyp in our study population.CONCLUSION Duodenal polyps are a rare finding on endoscopic examinations, though most of them are non-neoplastic in nature, vigilant examination under the microcope is required to rule out any neoplastic pathology and identify the risk of malignancy

## INTRODUCTION

**Figure 1:**
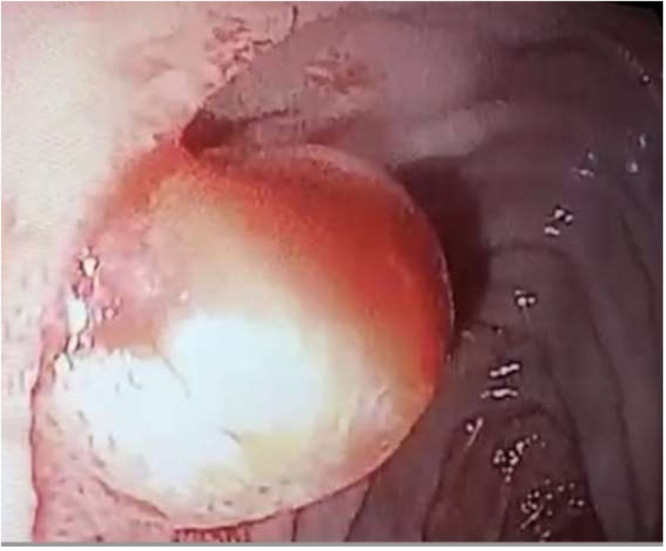
Endoscopic view of a pedunculated duodenal polyp

Duodenal polyps are not very common lesions in patients undergoing upper gastrointestinal (UGI) endoscopy, and the prevalence varies widely from 0.3-4.6% of cases^1,2^. Recent advances in endoscopic procedure and techniques have made precise excision of diminutive polyps (less than 2 cm in diameter) possible^3^ (Figure 1). Thus, the definite histopathological diagnosis of duodenal polyps has become easier despite them being rare in occurrence. Herein, we present the histopathological spectrum of duodenal polyps encountered over a period of 10 years in a tertiary care centre of North India and discusses the associated clinical and endoscopic findings.

## MATERIALS AND METHODS

The present study is a ten-year retrospective study from the year 2010 to 2020. Both adults and pediatric age groups were included. All the relevant records (i.e., demographics, clinical presentation, radiological findings, endoscopic findings, etc.) regarding the duodenal polyp biopsies submitted in the pathology department, were retrieved from the Institution's Hospital Intranet System (HIS). All the histology slides were re-examined and reviewed. A few old, faded slides were restained using regular hematoxylin and eosin (H&E) stain. Immunohistochemistry (IHC) and special stains were done wherever required. In few cases IHC was done manually and in others automated Ventana IHC system was used. The present study was an observational study with descriptive statistics, and all the collected data were continuous and categorical variables. Categorical values were represented as counts and relative frequencies whereas continuous variables were represented as medians and range. All the collected data were tabulated in a Microsoft excel sheet.

## RESULTS

Total 81 cases of duodenal polyp biopsies were studied out of all the submitted UGI endoscopic polyp cases. The age ranged from 13-81 years, and the median age was 48 years. The male (56): female (25) ratio was 2.2:1. Most of the patients presented with UGI symptoms such as abdominal pain, melena, dyspepsia, vomiting and weight loss; among all, abdominal pain was the most common finding, which was seen in 21 (25.9%) cases. The incidence of different kinds of duodenal polyps seen in the present study has been tabulated in Table 1. Most duodenal polyps were found to be of non-neoplastic origin, among which one showed evidence of granulomatous lesion while the other showed nonspecific inflammation. The adenomatous polyp was one of the most common among neoplastic causes, followed by neuroendocrine tumours. Among adenomatous polyps, four adenomatous polyps were found to be more than 3 cm in their largest dimension.

**Table 1:** Neoplastic and non-neoplastic duodenal polyps

	Diagnosis	Number of cases (n = 81)
Non neoplastic	Unremarkable mucosa or inflammatory polyp	32 (40%)
	Hyperplastic polyp	10 (12%)
	Brunner’s gland adenoma	08 (10%)
	Hamartomatous polyp	04(5%)
	Lipomatous polyp	01(1.2%)
Neoplastic	Adenomatous polyp	13 (16%)
	Neuroendocrine tumour	9(11%)
	NHL	01(1.2%)
	Gangliocyticparaganglioma	01(1.2%)
	GIST	01(1.2%)
	Adenocarcinoma	01(1.2%)

The endoscopic findings showed duodenal polyps were present either solitarily or as multiple polyps. Solitary polyps were found in 53 cases (65.4%), majority of which were present in the first part of the duodenum. The remaining 28 cases (34.6%) presented with multiple coexisting polyps in the duodenum or in the other parts of GI tract, among which the majority were found to coexist with gastric polyps (9 cases, 32%). Polyps were of variable sizes, ranging from 0.5 cm to 5 cm, had multilobulated appearance and majority (75.3%) were pedunculated. The distribution of the cases which showed multiple polyps in addition to duodenal polyps, has been tabulated in Table 2.

**Table 2:** The distribution of multiple duodenal polyps in the gastrointestinal tract including those restricted to duodenum itself

Site	N = 28
Gastric	9 (32%)
Duodenum	5 (18%)
Jejenum	3 (11%)
Colon	5 (18%)
Unspecified	6 (21%)

## DISCUSSION

To our best knowledge, it’s the first study in India, describing specifically the histopathological spectrum of duodenal polyps. In the present study, most polyps were non-neoplastic, and the majority displayed normal duodenal mucosa and inflammation on histopathological examination. Granulation tissue was noted in one case, and one case demonstrated reactive lymphoid follicle. One case among the non-neoplastic polyps had granulomatous lesion in association with ulcerated mucosa. Similar to the present study, the study done by Jung et al. showed that most of the non-neoplastic polyps had normal duodenal mucosa or histopathology suggestive of an inflammatory polyp^4^.

**Figure 2:**
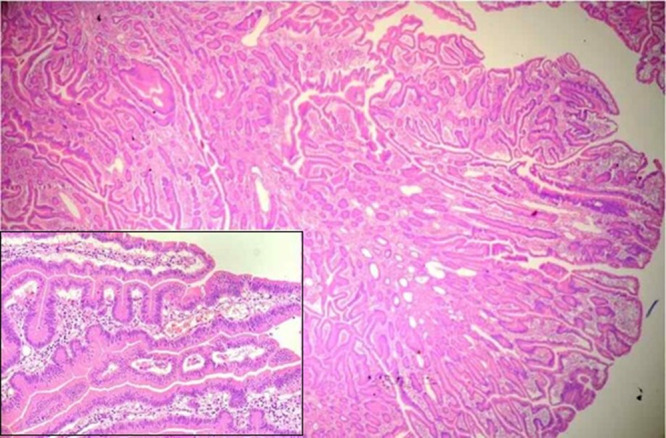
Hyperplastic polyp with elongated crypts, superficial serration and decreased goblet cells (H&E, main image 40x magnification and inset 100x magnification).

The hyperplastic polyp was the second most common non-neoplastic polyp. Unlike previous studies where duodenal polyps were mostly found in the second part of the duodenum, the most common site for the hyperplastic polyp in the present study was the first part of the duodenum^5^ (Figure 2). Hyperplastic polyps of the duodenum are rare and exhibit a histological appearance similar to microvesicular hyperplastic polyp of the large bowel. Though the pathogenesis is not clear, hyperplastic polyps are usually associated with Helicobacter pylori infection or other forms of gastroduodenal infections^5^.

**Figure 3:**
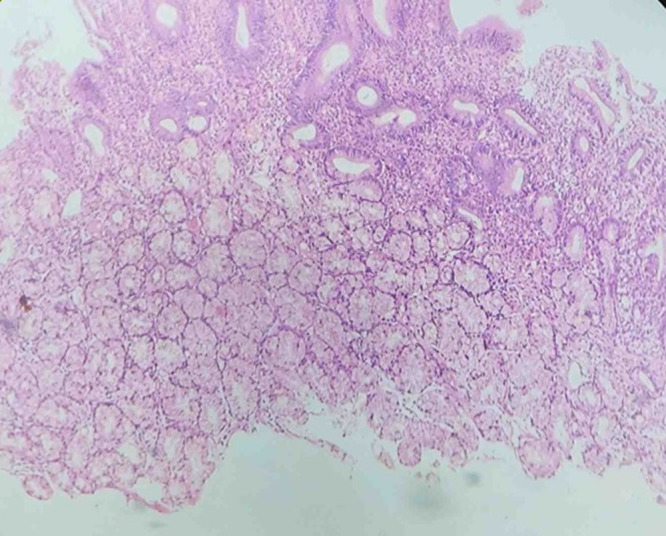
Brunner gland hyperplastic polyp displaying submucosal proliferation of Brunner’s gland (H&E; 40x magnification).

In the present study, Brunner’s gland hyperplasia mainly presented as a solitary polyp in the first part of the duodenum. Brunner’s gland hyperplastic nodules or polyps are characterised by lobular proliferation of hyperplastic glands in the submucosa and are commonly located in the duodenal bulb (Figure 3). They are usually associated with gastric acid hypersecretion, Helicobacter pylori infection, end stage renal disease (ESRD), uraemia and chronic pancreatitis^6,7^.

Four cases of Hamartomatous polyp were noted in the present study, of which two were associated with Peutz-jeghers (P-J) syndrome. All four cases presented with multiple polyps involving various parts of the GI tract. One of the P-J syndrome cases presented with malrotation along with duodenojejunal intussusception. In contrast, the other case was asymptomatic and had a family history of P-J syndrome. Hamartomatous polyps often display normal epithelial and stromal cells, which are arranged haphazardly and include juvenile polyps, Peutz-Jegher’s polyp, PTEN hamartomatous polyps and the associated polyposis syndrome. Peutz-Jegher’s polyposis syndrome is autosomal dominant in inheritance and characterized by STK11/LKB1 gene mutation^8,9^. Juvenile polyposis syndrome is associated with more than five juvenile polyps present anywhere along the entire GI tract along with a relevant family history. If clinical features are inconclusive, then identifying pathogenic variants of SMAD4 or BMPR1A is necessary for diagnosis. The PTEN hamartoma tumour syndrome (PHTS) encompasses four distinct syndromes associated with germline mutations of the PTEN tumour suppressor gene; this umbrella of syndromes includes the Cowden Bannayan-Riley-Ruvalcaba syndrome, Proteus syndrome, and Proteus-like syndrome^10,11^.

**Figure 4:**
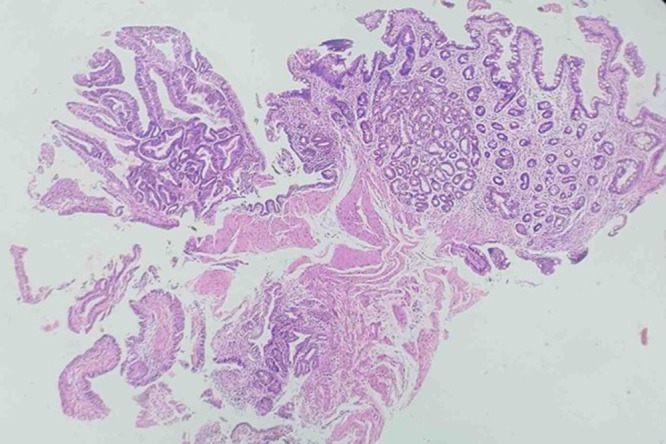
Villous adenomatous polyp, displaying finger-like projections (H&E; 40x magnification).

In the present study, adenomas were the most common among the neoplastic etiologies, followed by the neuroendocrine tumours. Most of the adenomatous polyps presented as sporadic solitary polyp (10 out of 13 cases) and were commonly located in the first part of the duodenum. Rest three presented as multiple adenomatous polyps. Various studies have described sporadic non-ampullary duodenal polyps as commonly sessile, solitary, and located in the second portion of the duodenum^12,13^. Contrary to the previous studies, our study showed the first part of the duodenum as the most common site for the sporadic nonampullary adenomatous duodenal polyps (Figure 4). One among the three cases of multiple adenomatous polyps was seen to be associated with familial adenomatous polyposis (FAP) syndrome. Multiple duodenal adenomatous polyps are usually found to be associated with genetic syndromes, including familial adenomatous polyposis (FAP), attenuated FAP, or MUTYH associated adenomatous polyposis (MAP). They are often sessile polyps with a predilection for the distal duodenum and periampullary region. Owing to their small size, they can be easily missed during upper GI endoscopy. However, with the aid of chromoscopic techniques, the number of detected polyps has increased considerably^14^. Among the adenomatous polyps, we had definite evidence of invasion in only one case, adenocarcinoma. Nine neuroendocrine tumour cases (NET) were diagnosed on biopsy, among which eight were of grade I and one was of grade II. Seven cases presented as solitary polyp. Two cases presented as multiple polyps of which one case had evidence of simultaneous gallbladder neuroendocrine tumour, and another had a coexistent stomach neuroendocrine tumour in addition to the duodenal polyps. On IHC, all the cases showed positivity for synaptophysin, chromogranin and CD56. Ki67 was <2% in all except one case of grade II tumour where it was more than 10%. The first part of the duodenum is the most common site for neuroendocrine duodenal polyps, and the present study had five cases with similar presentations. All of these tumours in the present study were non-functional in nature^15^. The WHO grading system for NET of GI tract was revised in 2017. It described a new subset of well-differentiated NENs, morphologically well-differentiated and often identical to grade 1 or grade 2 NET but have a high Ki-67 index (> 20%)^16^. Among the less commonly experienced entities, we experienced one case each of non-Hodgkin lymphoma associated polyp, gangliocytic paraganglioma associated polyp, lipomatous polyp and gastrointestinal stromal tumor (GIST) associated polyp. In the present study, gangliocyic paraganglioma was reported in a 68-year-old male patient at the D2-D3 junction. Gangliocytic paragangliomas (GP) are rare and predominantly found in the second part of the duodenum. They are usually benign tumours with very occasional evidence of lymph node metastasis. Clinical presentation varies from incidental findings on endoscopy to abdominal pain and upper gastrointestinal bleeding^17^. Histologically GP are triphasic and are composed of three cell types: (1) epithelioid cells, resembling well-differentiated neuroendocrine tumors or paraganglioma in both cytologic and architectural features; (2) spindled cells, resembling those seen in peripheral nerve sheath tumors; and (3) ganglion-like cells with characteristic abundant cytoplasm and prominent nucleoli. The cell types exhibit differential IHC staining patterns as well, with epithelioid cells and ganglion cells showing positivity for neuroendocrine markers along with progesterone receptor and pancreatic polypeptide positivity, while the spindled cells showed positivity for S100 and BCL2^18^. A single case of the lipomatous polyp was found in the 70-year-old post renal transplant patient who presented with multiple polyps in the second part of duodenum. Duodenal lipomas (DL) are extremely rare. The incidence of DL is so less that they are only limited to case reports. They are benign and slow-growing tumours. The peak incidence is seen in the seventh decade of life. The exact aetiology of DL remains unknown. It might be associated with embryonic displacement of adipose tissue, degenerative disease with disturbance of fat metabolism or dyslipidemia. Multiple DL is a very rare finding, and there are only 11 cases reported so far, including our case in the present study^19^.

## CONCLUSION

Our study specifically highlights histopathological spectrum of duodenal polyps. Duodenal polyps are rare finding on UGI endoscopic examinations. Most of them were benign in the present study and histologically majority of them displayed unremarkable mucosa or inflammatory pathology. Other than the above findings, the present study also noted hyperplastic polyp and adenomatous polyp most commonly in the first part of the duodenum, which is unlike to their common location (i.e., second part of duodenum). We also encountered one case each of multiple DL and GP in present study, respectively, both the entities are very rarely acknowledged. Among the polyps with neoplastic nature, adenomatous polyps were most commonly encountered in the present study. In near future we need more such studies where we can explore and know the possible variations that can be observed in the clinico-pathological profile of duodenal polyps.
